# Prevalence and correlates of malnutrition among children aged 6-59 months in the rural areas of Embu County, Kenya: a cross-sectional study

**DOI:** 10.11604/pamj.2026.53.126.48300

**Published:** 2026-03-13

**Authors:** Annah Muendi, Peter Ntoiti Kailemia, Daniel Nthiwa

**Affiliations:** 1 Department of Community Health, University of Embu, Embu, Kenya,; 2 Department of General Nursing, Meru University of Science and Technology, Meru, Kenya,; 3 Department of Biological Sciences, University of Embu, Embu, Kenya

**Keywords:** Malnutrition, nutritional status, associated factors, children aged 6-59 months, Kenya

## Abstract

**Introduction:**

childhood malnutrition remains a major public health challenge in developing countries, with poorly understood sustaining factors. This study assessed the nutritional status and identified factors associated with malnutrition among children aged 6-59 months in the rural areas of Embu County, Kenya.

**Methods:**

a cross-sectional study was conducted involving 388 children aged 6-59 months from 330 households between December 2023 and January 2024. Structured questionnaires were used to collect data. Anthropometric measurements were taken from the children, converted into height for age (HAZ), weight for age (WAZ), and weight for height (WHZ) Z scores, and compared with the World Health Organization (WHO) standard measurements to assess the nutritional status. Logistic regression analyses were performed to evaluate factors associated with the various malnutrition indicators.

**Results:**

the overall prevalence of malnutrition was 49.7%, with undernutrition being 40.5% and overnutrition 15.7%. For stunting, it was 30.4%, underweight 14.4%, wasting 13.7%, and overweight/obese 15.7%. In addition, the prevalence of coexisting malnutrition ranged from 3.6% to 6.7%. The overall malnutrition prevalence differed by study sites and was also high among the partially immunized children in comparison with fully immunized children (Adjusted Odds Ratio [AOR] = 1.67; 95% CI: 1.03 - 2.72). Overnutrition was significantly associated with a child´s immunization status, household food insecurity, and the caregiver's time for child food preparation. Child´s age, gender, birthweight, anthelmintic drug administration, and child´s food allocation practices were associated with underweight. Predictors of overweight/obese included immunization status, birth weight, and household food insecurity status. Children from severely food-insecure households were more likely to be overweight/obese (AOR = 2.49; 95% CI: 1.07-5.81) than those from food-secure households. Stunting was associated with child´s food allocation practice.

**Conclusion:**

the high burden of malnutrition, the coexistence of undernutrition and overnutrition suggest the existence of diverse drivers within the same context. Tailored multicomponent interventions targeting different contextual levels may reduce the malnutrition burden in the study area.

## Introduction

Malnutrition significantly affects children´s health and growth. Childhood malnutrition may manifest in numerous forms including stunting, wasting, underweight, and overweight [1]. Approximately, 149 million children are stunted, 45 million wasted, and 38.9 million are overweight globally [1]. In 2020, there were almost 45.4 million children aged 6-59 months who were wasted, and 13.6 million severely wasted [2]. Africa has a higher rate of malnutrition compared to other regions with a stunting prevalence of 39.4% [3]. In sub-Saharan Africa, high rates of malnutrition are found in South Sudan, Nigeria, and Somalia [4]. Undernutrition in sub-Saharan Africa stands at 35.5%, with a higher prevalence recorded in Somalia (51.3%) compared to Kenya (34.5%) while wasting and underweight prevalence estimates are 6.0% and 12.8%, respectively [5]. North African nations have 23.5% stunting, 7.9% wasting, and 12.9% underweight [6]. However, many studies have reported on undernutrition among children aged 6 - 59 months with limited attention to emerging child overnutrition [7-9].

Malnutrition may disrupt a child´s intellectual and physical growth problems later in life. Infectious illnesses and mortality rise with wasting while childhood overweight and obesity may cause cardiovascular disorders. Malnutrition also impacts the economic status of a country. For instance, US$3 trillion is used per annum due to undernutrition [5]. In Kenya, stunting prevalence amongst children aged 6-59 months is estimated to be 26.2% with malnutrition contributing to approximately 35% of mortality in this population [10]. Childhood malnutrition contributes to 35% of the disease burden and 11% of disability-adjusted life years [11]. It results from individual, household, societal and environmental variables [12]. Intra-household food distribution such as food allocation is linked to child nutritional outcomes [13,14]. In resource limited environments, caregiver time affects feeding frequency, meal preparation and quality of diet [15]. Preventive health measures such as immunization and deworming result in decreased nutritional stress in infections and are always associated with better child developmental outcomes [16,17]. The quality and diversity of diet also affect childhood malnutrition [18].

Despite the presence of nutrition interventions in Kenya, childhood malnutrition prevalence in the informal settlements and rural areas remains high with limited study. There is also inadequate context-specific data on factors associated with high malnutrition rates in Embu County. Thus, this study assessed nutritional status and identified factors associated with malnutrition among children aged 6-59 months in the rural areas of Embu County, Kenya. Our findings will guide the design of targeted strategies to mitigate childhood malnutrition.

## Methods

**Figure 1:**
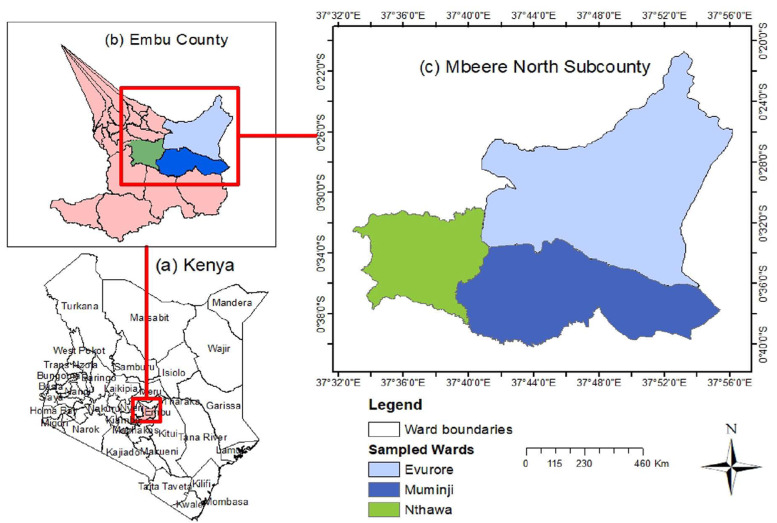
map of the study area: A) Embu County in Kenya; B) location of surveyed wards in Embu County; C) Mbeere North Sub-County

**Study area:** this study was implemented in Mbeere North Sub-County in Embu County, Kenya. The study site has an estimated area of 777 km^2^ with an approximate population of 108,881 people [19]. The study was conducted in Nthawa, Muminji and Evurore wards (Figure 1). The area was selected for this study due to food insecurity and high malnutrition risks linked to prolonged droughts experienced in the region [20]. The area also relies on rain-fed crop farming, and livestock production as the primary socioeconomic activities. Nine representative villages, three for each ward, were purposively selected for the study. The villages were selected based on the number of children aged 6-59 months and physical accessibility for data collection.

**Study population, study design and sample size estimation:**this study targeted children aged 6-59 months and their caregivers who were residents in the study area for at least six months preceding the survey. This study excluded children aged 6-59 months who were critically ill at the time of data collection and/or had physical deformities of the limb or spine. Children of caregivers who did not provide written informed consent to participate were also excluded in the study. Data were collected through a cross-sectional study between December 2023 and January 2024. The sample size (n) for this study was determined using the Cochran formula;


$$n=\frac{1.96^{2}xpx\left(1-p\right)}{d^{2}}$$

Where p denotes the estimated proportion of the study population with malnutrition and d^2^is the maximum allowable error (0.05) [21]. The study assumed a priori prevalence of malnutrition (p) to be 50% due to limited information on malnutrition in the study area. The estimated sample size for the study was 384 children. However, 388 children matched to 330 caregivers were included in the study. The number of children included was allocated proportionately between wards based on the number of children meeting the inclusion criteria. The percentage distribution of the children by wards was 31.70% (Muminji), 32.99% (Evuvore), and 35.31% (Nthawa).

**Selection of households and children:** a sampling frame was built on community health registers of Community Health Promoters (CHPs) within the chosen villages comprising households with at least one child aged between 6 and 59 months. A list of households with children/caregivers meeting the inclusion criteria was then compiled per village in the selected wards. The households were then selected randomly using the simple random sampling technique from the compiled sampling frame. All children aged 6 and 59 months in the identified households were eligible for inclusion in the study. If a household was not available after two consecutive visits, it was treated as non-responding and substituted with another randomly-selected household from the same village. Potential selection bias was considered because daytime household availability may over-represent certain caregiver profiles, especially caregivers who did not participate in formal employment, which might also affect the observed practices in caregiving.

**Data collection:** data were collected using individual and household customized structured questionnaires that were designed using the KoboCollect application. Before data collection, CHPs enumerators were trained for three days on the objectives of the study, informed consent, questionnaire administration, and anthropometric measurements. Data collection tools were pre-tested using 20 caregivers to child pairs within the study area in the villages not selected for the main study. The household questionnaire with adapted questions to the local context from the Demographic and Health Surveys (DHS), Food and Agriculture Organization (FAO) and Food and Nutrition Technical Assistance (FANTA) measurement tools [22,23] was administered in each household to collect data on household socio-demographics, children´s dietary diversity, household food insecurity and environmental health factors related to malnutrition including waste disposal, type of toilet, source of drinking water and sanitation. The household food security was determined using the Household Food Insecurity Access Scale (HFIAS), a 30-day-recall period that included nine occurrence questions and nine frequency-of-occurrence questions which were asked as a follow-up to establish how often each condition occurred [23]. The maximum possible HFIAS score is 27 with higher scores indicating greater levels of food insecurity. This questionnaire was also used to assess the caregiver´s knowledge on child nutrition and care practices. The caregivers were interviewed using the 24-hour dietary recall to assess child feeding practices including food allocation practices in the family and the variety of foods given to the child in the preceding 24 hours. The dietary diversity was calculated per child based on the nine food groups recommended by FAO [24]. The maximum possible score was nine, showing that the child had consumed from all the nine food groups at least once. Children who consumed four or more food groups were categorized as having adequate dietary diversity. The individual questionnaire had questions adapted from the United Nations Children Fund (UNICEF) anthropometry measurement tool [25] and was used to record child-related data including gender, age, birthweight, birth order, immunization status, vitamin A supplementation and deworming status; and anthropometric measurements (height and weight). The anthropometric measurements were recorded following the WHO standard guidelines [25]. All anthropometric measurements were taken thrice and the average used for analysis.

**Data analyses:** data collected using the questionnaires were merged and cleaned up to have complete data before analyses. The height-for-age (HAZ), weight-for-age (WAZ) and weight-for-height (WHZ) Z scores were calculated using the WHO Anthro software [26] and interpreted with reference to the WHO standard measurements [16] to identify the different forms of malnutrition using cut-off values as follows: stunting (HAZ < -2 SD), underweight (WAZ < -2 SD), wasting (WHZ < -2 SD), overweight (WHZ > +2 SD), and obesity (WHZ > +3 SD). Children with the above overall forms of malnutrition were further classified into moderate or severe categories for each malnutrition form. Moderate and severe stunting were defined as HAZ <-2 SD and HAZ <-3 SD, respectively. Children with WAZ scores <-2 SD and <-3 SD were respectively categorized as moderately or severely underweight while for wasting, those with WHZ scores <-2 or -3 SD were further classified as moderately and severely wasted, respectively.

Descriptive statistics were calculated using the R statistical software, version 3.6.3 [27]. These included the calculation of the overall prevalence of malnutrition, determined as the percentage of children with any form of undernutrition and/or overnutrition combined. The prevalence estimates of children with undernutrition combined, stunting, underweight, wasting and overweight/obese and those with concurrent multiple forms of malnutrition were also computed and stratified by categorical independent variables. These estimates were obtained through cross-classification tables using the stats package [28] with the χ^2^test being included to evaluate the relationships between the various forms of malnutrition and the categorical factors. The 95% confidence intervals were computed using the multinormCI function in the DescTools package [29].

Further analyses of independent factors associated with the binary dependent variables (underweight/normal, and stunted/normal) were conducted by fitting the data to univariable logistic regression models using the glm function in stats package. Independent factors with p values ≤0.05 from the univariable models for each malnutrition form were selected to fit corresponding maximal multivariable models. These were then reduced through stepwise backward deletion approach to identify the most parsimonious final models with the lowest Akaike´s information criterion (AIC) and significant covariates. The covariates included in the models were selected based on the conceptual framework and the available literature on the factors associated with malnutrition. The dependent variable based on WHZ cut-off values with three levels (normal, wasted and overweight/obese), univariable and multivariable multinomial logistic regression models were fitted using the multinom command in nnet package [20] following the same approach as for the univariable and multivariable models explained above. Final models fit adequacy and interactions of the covariates was checked using the likelihood ratio test (LRT) and plots of residuals versus fitted values.

**Ethical considerations:** the National Commission for Science, Technology and Innovation (NACOSTI), granted permission to conduct this study (reference number: 977758). Ethical approval was provided by the Chuka University Institutional Ethics Review Committee (Approval number: NACOSTI/NBC/AC-0812). Participation in the study was voluntary and the caregivers of the children provided written informed consents to participate in the research after they were adequately informed about the study.

## Results

**Descriptive results:** Table 1 summarizes the child, household, sociodemographic and environmental results of the study participants. Out of 388 children included in the study, 199 (51.29%) were males and 189 (48.71%) females. Overall, the median number of children aged 6-59 months in the households was 1 (range: 1-3). The median birthweight of the children was 3.0 kg (range: 1-5 kg) while the median weight of the children during the study was 12.0 kg (range; 6-25kg). The median age of the children was 31 (range: 6-59) months. The median family size was 4 (range: 2-14) with the median age of the household heads and caregivers were 33 (range: 18-75) and 28 (range: 18-67) years, respectively.

**Table 1:** child, household, sociodemographic, and environmental characteristics of the study participants

Variable	Category	n (%)
**Child characteristics (N=388)**		
Gender	Male	199 (51.29)
	Female	189 (48.71)
Age group (in months)	6-23	133 (34.28)
	24-47	170 (43.81)
	48 and above	85 (21.91)
Birth weight	Below 2.5kg	54 (13.92)
	≥2.5kg	334 (86.08)
Immunization status	Fully	292 (75.26)
	Partially	96 (24.74)
Anthelmintic drug administration	No	31 (7.99)
	Yes	354 (91.95)
	Don't know	3 (0.77)
Child given vitamin A supplementation	No	11 (2.80)
	Yes	375 (96.60)
	Don't know	2 (0.50)
**Household and sociodemographic characteristics (N=330)**		
Household head gender	Male	192 (58.18)
	Female	138 (41.82)
Age of household head	10-24	40 (12.27)
	25-34	141 (43.25)
	35-44	104 (31.90)
	45-54	27 (8.28)
	≥55	14 (4.29)
Marital status of the household head	Single	95 (28.79)
	Married	232 (70.30)
	Windowed	3 (0.91)
Occupation of the household head	Farming	224 (67.88)
	Business	43 (13.03)
	Employed	18 (5.46)
	Not employed	32 (9.70)
	Others^a^	13 (3.94)
Gender of the caregiver	Male	5 (1.52)
	Female	325 (98.49)
The education level of the caregiver	No formal education	9 (2.73)
	Primary	212 (64.24)
	Secondary	89 (26.97)
	Tertiary	20 (6.06)
Age of the caregiver	15-19 years	13 (3.95)
	20-29 years	175 (53.19)
	30-39 years	107 (32.52)
	40-49 years	26 (7.90)
	≥ 50 years	8 (2.43)
Number of children from study sites (wards)	Evurore	128 (33.00)
	Muminji	123 (31.70)
	Nthawa	137 (35.31)
**Environmental factors (N=330)**		
Type of toilet	No latrine	9 (2.73)
	Pit latrines	313 (94.85)
	Flushables	8 (2.42)
Waste disposal	Open air	185 (56.06)
	Refined pit	111 (33.64)
	Compost pit	25 (7.58)
	Burning	9 (2.72)
Source of drinking water	Tap water	153 (46.36)
	Bore hole	11 (3.33)
	River	88 (26.67)
	Other sources^b^	78 (23.63)
Hand washing practice	Always	170 (51.52)
	Sometimes	160 (48.49)

n: number of participants; %: percentage; kg: kilograms; ≥: greater than or equal to; ^a^Other occupations included: masonry, casual, mechanic; ^b^: other sources of drinking water included: community water points, open wells, irrigation water, and rain water

**Caregiver´s knowledge on child nutrition and care practices:** the caregiver´s knowledge on the right age of starting complementary feeding revealed that the majority of caregivers (59.09%) cited complementary feeding should begin at 24 months (Table 2). Mushed food (40.98%) was the most common food introduced to the children. Only 36.34% of caregivers breastfed their children. Based on the 24-hour dietary recall, most of the children had inadequate dietary diversity (84.28%) with 57.73% having the accepted minimum meal frequency. The majority of the households (74.24%) were food insecure.

**Table 2:** caregiver’s knowledge on child nutrition and care practices among the study participants

Variable	Category	n (%)
Caregiver's knowledge on right age of starting complementary feeding	Until the child stopped breastfeeding	6 (1.82)
	6-11 months	2 (0.61)
	12-23 months	79 (23.94)
	Less than 6 months	12 (12.12)
	24 months	195 (59.09)
	Don't know	8 (2.42)
Reason for complementary feeding	Insufficient breast milk	322 (83.42)
	Other reasons	12 (3.11)
	Don't know	52 (13.47)
Breastfeeding status	No	247 (63.66)
	Yes	141 (36.34)
Method of food preparation for the child	With adult food	184 (47.67)
	Separate	166 (43.01)
	Both	36 (9.33)
Food allocation among children	Share meals with the whole family	10 (2.58)
	Individual plate	373 (96.13)
	Share meals according to age groups	5 (1.29)
Dietary diversity of the child (24-hour recall)	Inadequate	327 (84.28)
	Adequate	61 (15.72)
Household food consumption score	Poor	23 (6.97)
	Borderline	28 (8.49)
	Acceptable	279 (84.55)
Household food security status	Food secure	89 (25.52)
	Mild	74 (19.07)
	Moderate	132 (34.02)
	Severe	83 (21.40)

n: number of participants; %: percentage

**Nutritional status of the children:** in total, 49.7% (95% CI: 44.8-55.1) of the children had at least one form of malnutrition while the overall prevalence of undernutrition was 40.5% (95% CI: 35.6-45.6). Overall, 30.4% (95% CI: 26.0-35.3) of the children were stunted. In further analysis, 47.5% (95% CI: 39.0-57.2) of these children were moderately stunted while 52.5% (95% CI: 44.1-62.3) were severely stunted. The percentage of underweight children was 14.4% (95% CI: 11.1-17.8); majority of them (62.5%, 95% CI: 51.8-76.5) were moderately underweight while 37.5% (95% CI: 26.8-51.5) were severely underweight. Furthermore, 13.7% (95% CI: 9.3-18.2) of the children had wasting whereby, 69.8% (95% CI: 58.5-82.3) were moderately wasted versus 30.2% (95% CI: 18.9-42.7) who were severely wasted. Also, 15.7% (95% CI: 11.3-20.3) of the children were overweight/obese. Of these children, 55.7% (95% CI: 44.3-69.0) were obese while 44.3% (95% CI: 32.8-57.6) were overweight. Children with concurrent undernutrition and/or overnutrition indicators were estimated at 3.9% (95% CI: 0.0-8.9) for stunted, underweight and wasted, 6.7% (95% CI: 2.1-11.8) for stunted and underweight, 3.6% (95% CI: 0.0-8.7) for underweight and wasted, and 6.4% (95% CI: 1.8-11.5) for stunted and overweight/obese.

The distribution of the prevalence estimates of the various malnutrition manifestations among children stratified by categorical factors is shown in Table 3. More male children were significantly underweight compared to females. A higher number of children aged between 24 and 47 months were both underweight and wasted relative to those in other age categories, while those aged between 6 and 23 months were more overweight than the other age groups. Child birthweight was a significant determinant of underweight, wasting and overweight. Children with low birthweight (<2.5 kg) were associated with being more underweight and wasted compared to normal birthweight (≥2.5 kg) and vice versa for overweight. Most partially immunized children had at least one of the multiple indicators of malnutrition compared to those fully immunized. The child´s food allocation practice in the household was significantly associated with underweight and stunting.

**Table 3:** distribution of the prevalence estimates of the various forms of malnutrition among children aged 6-59 months by categorical factors that were significantly associated with at least one of the dependent variables

Variable and category*	N	Overall malnutrition % (95% CI)	χ^2^ p-value	Undernutrition % (95% CI)	χ^2^ p-value	Underweight % (95% CI)	χ^2^ p-value	Stunted % (95% CI)	χ^2^ p-value	Wasted % (95% CI)	Overweight/obese % (95% CI)	χ^2^ p-value
**Gender**												
Female	189	46.0 (39.2 -53.7)	0.154	37.6 (30.7 - 44.7)	0.257	10.0 (6.3-14.3)	0.017	27.5 (21.7-34.3)	0.226	12.2 (6.3-18.5)	14.8 (9.0-21.2)	0.579
Male	199	53.3 (46.2 - 60.4)		43.2 (36.7 - 50.7)		18.6 (13.6-24.0)		33.2 (26.6-39.8)		15.1 (9.0-21.7)	16.6 (10.6-23.2)	
**Age group**												
6-23 months	133	54.9 (46.6 -63.8)	0.096	40.6 (32.3 - 49.2)	0.230	13.5 (8.3-19.1)	0.039	32.3 (24.8-40.6)	0.804	11.3 (3.8-19.7)	23.3 (15.8-31.7)	0.003
24-47 months	170	50.6 (42.9 - 58.2)		44.1 (37.1 - 52.2)		18.8 (13.5-24.8)		30.2 (23.5-37.2)		18.8 (12.4-25.8)	11.2 (4.7-18.1)	
≥48 months	85	40.3 (30.6 - 51.3)		32.9 (23.5 - 43.2)		7.1 (3.5-12.9)		28.2 (20.0-38.6)		7.1 (0.0-15.7)	12.9 (5.9-21.5)	
**Birth weight**												
Below 2.5 kg	54	49.4 (44.0 - 55.1)	0.738	48.1 (35.2 - 61.5)	0.215	24.1 (14.8-36.3)	0.030	31.5 (20.4-44.3)	0.854	22.2 (11.1-33.5)	5.6 (0.0-16.8)	0.023
≥ 2.5 kg	334	51.8 (38.9 - 65.2)		39.2 (34.1 - 44.8)		12.9 (9.6-16.4)		30.2 (25.4-35.4)		12.3 (7.5-17.1)	17.4 (12.6-22.2)	
Child given anthelmintic drugs in the past six months*												
No	31	51.6 (35.5 - 69.5)	0.816	49.4 (32.3 - 66.3)	0.352	32.3 (19.4-50.4)	0.003	32.3 (19.4-50.4)	0.814	22.6 (9.7-40.4)	9.7 (0.0-27.5)	0.260
Yes	354	49.4 (44.1 - 54.8)		39.8 (34.7 - 45.2)		12.7 (9.6-16.2)		30.2 (25.4-35.1)		13.0 (8.5-17.8)	16.1 (11.6-20.9)	
**Study sites (wards)**												
Nthawa	137	40.9 (32.8 - 49.5)	0.015	34.3 (27.0 - 42.9)	0.181	14.8 (9.3-20.9)	0.857	32.8 (25.0-41.2)	0.662	14.1 (6.3-22.3)	19.5 (11.7-27.7)	0.252
Muminji	123	50.4 (42.3 - 60.1)		44.7 (36.6 - 54.3)		13.0 (8.1-19.1)		30.9 (23.6-39.7)		16.3 (8.9-25.3)	16.3 (8.9-25.3)	
Evurore	128	58.6 (50.8 - 68.0)		43.0 (34.4 - 51.7)		15.3 (10.2-21.6)		27.7 (20.4-35.2)		10.9 (5.1-18.2)	11.7 (5.8-19.0)	
**Immunization status**												
Fully	291	46.2 (40.4 - 52.2)	0.016	39.0 (33.6 - 45.0)	0.319	14.0 (10.3 - 17.9)	0.702	29.5 (24.3 - 34.8)	0.473	13.0 (8.2 - 17.8)	12.3 (7.5 - 17.1)	0.003
Partially	96	60.4 (51.0 - 70.6)		44.7 (35.4 - 55.4)		15.6 (9.4 - 22.9)		33.3 (25.0 - 43.6		15.6 (6.3 - 26.0)	26.0 (16.7 - 3 6.4)	
**Child??s food allocation practice**												
Share meal with other family members	10	70.0 (50.0 - 99.7)	0.395	39.9 (34.8 - 45.0)	0.104	13.9 (10.7 - 17.5)	0.044	29.5 (24.9 - 34.3)	0.020	13.9 (9.7 - 18.7)	15.2 (11.0 - 20.1)	0.571
Individual plate	373	49.3 (44.2 - 54.7)		70.0 (50.0 - 99.7)		40.0 (20 - 74.5)		70.0 (50.0 - 99.7)		10.0 (0 - 39.1)	20.0 (0 - 49.1)	
Share meal among age groups	5	40.0 (20.0 - 91.2)		20.0 (0 - 50.5)		0.0 (0 - 33.1)		20.0 (0 - 50.5)		0.0 (0 - 51.2)	40.0 (20 - 91.2)	
**Caregiver has enough time to prepare child’s food**												
No	24	66.7 (50.0 - 85.1)	0.087	50.0 (33.3 - 71.8)	0.326	12.5 (4.2 - 26.4)	0.780	45.8 (29.2 - 67.4)	0.090	4.2 (0.0 - 25.3)	37.5 (20.8 - 58.6)	0.007
Yes	364	48.6 (43.4 54.0)		39.8 (34.9 - 45.2)		14.6 (11.3 - 18.2)		29.4 (24.7 - 34.1)		14.3 (9.9 - 19.0)	14.2 (9.9 - 19.0)	

* HFIAS: household food insecurity access scale; CI: confidence interval; n: number of children in each category; χ^2^: Chi square test

### Factors associated with the various forms of malnutrition

**Univariable results:** the univariable models are summarized in Table 4. These results showed that children´s age, study sites (wards), and immunization status were statistically associated with the overall prevalence of malnutrition. Undernutrition was only associated with hand washing practice among the independent factors analyzed. Similarly, the child´s food allocation practice in the household was the only significant factor related to stunting. Children´s age, immunization status, HFIAS, and whether the caregiver had enough time to prepare the child´s food were identified as significant determinants of overweight/obese. From the multinomial models, none of the evaluated factors were significantly associated with wasting. Child gender (male), birthweight, lack of deworming, and child´s food allocation practice in the household were associated with underweight.

**Table 4:** univariable results depicting factors found to be significantly associated with the various forms of malnutrition among children aged 6-59 months

Malnutrition forms	Variable	Category	Odds ratio (95% CI)	P value
Overall malnutrition	Age group	6-23 months	Ref.	
		24-47 months	0.84 (0.53 - 1.33)	0.457
		≥48 months	0.54 (0.32 - 0.95)	0.033
	Study sites (wards)	Nthawa	Ref.	
		Muminji	1.47 (0.90 - 2.41)	0.124
		Evurore	2.04 (1.30 - 3.35)	0.004
	Immunization status	Fully	Ref.	
		Partially	1.78 (1.11 - 2.85)	0.017
Undernutrition	Hand washing practice	Always	Ref.	
		Sometimes	1.71 (1.14 - 2.59)	0.010
Stunting	Child's food allocation practice	Individual plate	Ref.	
		Share meal with other family members	5.58 (1.52 - 26.24)	0.014
		Share meal among age groups	0.59 (0.03 - 4.10)	0.647
Underweight	Gender	Female	Ref.	
		Male	2.04 (1.14 - 3.77)	0.018
	Birth weight	≥ 2.5 kg	Ref.	
		Below 2.5 kg	2.15 (1.03 - 4.24)	0.033
	Child given anthelmintic drugs in the past six months	Yes	Ref.	
		No	3.27 (1.40 - 7.25)	0.004
	Food allocation practice	Individual plate	Ref.	
		Share meal with other family members	4.12 (1.02 - 1.49)	0.033
		Share meal among age groups	1.07 (0.00 - 2.37)	0.983
Overweight	Age group	6-23 months	Ref.	
		24-47 months	0.45 (0.23 - 0.84)	0.013
		≥48 months	0.45 (0.21 - 0.97)	0.041
	Immunization status	Fully	Ref.	
		Partially	2.70 (1.50 - 4.87)	<0.001
	HFIAS*	Food secure	Ref.	
		Mild	1.53 (0.60 - 3.88)	0.368
		Moderate	1.50 (0.65 - 3.39)	0.336
		Severe	2.49 (1.08 - 5.74)	0.032
	Caregiver has enough time to prepare child's food	No	Ref.	
		Yes	0.31 (0.13 - 0.76)	0.010

* HFIAS: household food insecurity access scale; Ref: reference category; OR: odds ratio; CI: confidence interval

**Multivariable results:** from the multivariable results shown in Table 5, the overall prevalence of malnutrition varied significantly by the study sites. More children in the Evurore ward had at least one form of malnutrition compared to those in the Nthawa region. In contrast, this pattern was not observed between Muminji and Nthawa wards. Partially immunized children were also 1.67 times more likely to develop any form of malnutrition compared to those fully immunized.

**Table 5:** results from the multivariable models showing factors associated with the various forms of malnutrition among children aged 6-59 months

Malnutrition forms	Variable	Category	Odds ratio (95% CI)	P-value
Overall malnutrition	Study sites (wards)	Nthawa	Ref.	
		Muminji	1.53 (0.93 - 2.51)	0.094
		Evurore	1.91 (1.17 - 3.15)	0.010
	Immunization status	Fully	Ref.	
		Partially	1.67 (1.03 - 2.72)	0.037
Underweight	Gender	Female	Ref.	
		Male	2.37 (1.28 - 4.54)	0.008
	Birth weight	≥ 2.5 kg	Ref.	
		Below 2.5 kg	2.19 (0.10 - 4.60)	0.042
	Child given anthelmintic drugs in the past six months	Yes	Ref.	
		No	4.74 (1.85 - 11.97)	<0.001
	Food allocation practice	Individual plate	Ref.	
		Share meal with other family members	10.04 (2.22 - 43.83)	0.002
		Share meal among age groups	0.00*	0.989
	Age group	6-23 months	Ref.	
		24-47 months	2.21 (1.09 - 4.71)	0.032
		≥48 months	0.70 (0.23 - 1.96)	0.513
Overweight alternative model 1	Immunization status	Fully	Ref.	
		Partially	2.65 (1.47 - 4.50)	0.001
	Birth weight	≥ 2.5 kg	Ref.	
		Below 2.5 kg	0.32 (0.09 - 1.10)	0.070
Overweight alternative model 2	Immunization status	Fully	Ref.	
		Partially	2.76 (1.52 - 5.01)	<0.001
	Caregiver has enough time to prepare child food	No	Ref.	
	
		Yes	0.30 (0.12 - 0.74)	0.009
Overweight alternative model 3	Immunization status	Fully	Ref.	
		Partially	2.71 (1.50 - 4.91)	0.001
	HFIAS*	Food secure	Ref.	
		Mild	1.53 (0.60 - 3.91)	0.378
		Moderate	1.46 (0.64 - 3.35)	0.369
		Severe	2.49 (1.07 - 5.81)	0.035

* : the 95% CI for this estimate could not be computed; **HFIAS: household food insecurity access scale; Ref: reference category; AOR: adjusted odds ratio

For underweight, more male children were significantly underweight compared to females. Children with low birthweight (<2.5 kg) had increased odds of being underweight compared to those with an ideal birthweight of more than or equal to 2.5 kg. Furthermore, children who had not been given anthelmintic drugs in the six months preceding the study were 4.74 times more likely to be underweight compared to those dewormed. Children who shared meals with other family members were found to have significantly higher odds of being underweight compared to those served food on individual plates. Children between the age of 24-47 months had 2.21 odds of being underweight compared to those between 6-23 months.

Concerning overweight/obese, three final alternative models were fitted to the data based on the combinations of different factors (Table 5). A final model with immunization status and birthweight as forced fixed effect was considered due to a lower AIC value (620.55) than a model with only immunization as the fixed effect with an AIC value of 624.45. These models showed that partially immunized children were more likely to be overweight than fully immunized children. Caregivers who had enough time to prepare their food had significantly lower odds of having overweight children than those who did not have enough time to prepare their food. Children from households with severe HFIAS had 2.49 odds of being overweight compared to those from food-secure households.

## Discussion

This study provides evidence of malnutrition among children aged 6-59 months with nearly half (49.7%) of the children in Embu County having at least one form of malnutrition. This indicates a major public health problem in the study area and necessitates immediate interventions. The results also confirm the co-occurrence of over and undernutrition (double burden of malnutrition) an issue of concern in many developing countries undertaking dietary transformations [30]. The overall prevalence of malnutrition varied by study sites, with the highest prevalence in Evurore compared to Nthawa, which might be attributed to the socioeconomic, environmental, and cultural differences in prevalence of malnutrition and access to healthcare across the study sites. Partially immunized children were more likely to have malnutrition than fully immunized children. This finding supports the evidence that vaccination helps prevent infections that may accelerate the pathogenesis of malnutrition among children [31,32]. Children aged between 6 and 23 months were more likely to have malnutrition than those aged ≥48 months. This age marks the transition period from exclusive breastfeeding to complementary feeding; hence, improper feeding practices and insufficient dietary diversity can make the body more vulnerable to nutrients. This finding emphasizes the importance of caregiver practices in child nutrition outcomes [13].

This study revealed a higher prevalence of undernutrition (40.5%) compared to the pooled prevalence of 33.3% reported in East Africa [14]. This implies that a substantial proportion of these children are at risk of developing severe and long-lasting consequences attributable to undernutrition [15] including elevated risk of acquiring infections, especially diarrhoea and pneumonia [16]. Whilst undernutrition is linked to multiple factors, increased odds of undernutrition were found among children who washed their hands sometimes relative to those who always washed their hands, as reported in other studies [17,18]. This finding could be related to exposure to infectious agents including diarrhoea-causing pathogens which supports the integration of water, sanitation, and hygiene (WASH) interventions with nutrition programs [33,34]. Stunting was the most prevalent (30.4%) form of undernutrition, consistent with other studies [18,32]. Although it was higher than Kenya´s national average of 26% [11], lower estimates have been reported by other studies, for example, in North African nations (23.5%), Ethiopia (48.9%) [35], Burundi (53%) [14], and Kenya (46.0%) [11]. Children who shared meals with family members were more likely to be stunted compared to those served on individual plates possibly due to limited nutrient intake [35,36].

The prevalence of underweight children (14.4%) was comparable to the average of 15.9% in sub-Saharan Africa [37] and North African nations (12.9%) [6,37]. Nevertheless, other studies reported higher estimates; Burundi (27.6%) [37,36] and Nigeria (21.73%) [30] and lower estimates in Kenya (9.8%), Uganda (7.6%), Tanzania (11.4), and Rwanda (7.7%) [37]. Child gender (males) and age (above 24 months) were strongly associated with underweight consistent with another study [38] possibly reflecting biological vulnerabilities or gender-based differences in care practices. Low birthweight also significantly increased underweight risk, affirming its role as a predictor of suboptimal weight gain [38]. This association underscores the importance of maternal nutrition and antenatal care in preventing child malnutrition [39]. Lack of deworming medication was also linked with underweight which is connected to parasite infections. This affects children´s nutrition and growth causing malabsorption of nutrients, impairing digestion, affecting child´s appetite and food intake [40]. The observation that children who shared food among family members had increased odds of being underweight compared to those served food on individual plates is related to suboptimal feeding practices.

The prevalence of wasting (13.7%) was higher than in Kenya (4.2%) [10], North African nations (7.9%) [6] and Nigeria (6.92%) [30]. Based on the χ^2^ test, low birthweight (<2.5kg), age (24-47-months) and partial immunization status were significantly associated with wasting. Low birthweight children have lower nutritional reserves and immune systems hence are more vulnerable to early childhood wasting compared to birthweight of ≥2.5kg [41]. Children aged between 24 and 47 months were more wasted than those aged 6-23 months, probably due to increased activity, nutritional needs during fast growth and development, dietary deficiencies linked to poor weaning practices and improper complementary feeding [13]. This age category is also vulnerable to infections like pneumonia and diarrhoea due to increased environmental interactions [14]. Children of caregivers who had more time to prepare their meals were more wasted than those of caregivers who did not have enough time. This implies that time is not sufficient to guarantee adequate nutrition; education and access to food are critical in child wasting prevention.

The prevalence of overnutrition (15.7%) was higher than the national estimate of 4.1% [10]. However, it was comparable to a study in Egypt (17%) [42]. Overweight/obese was strongly associated with partial immunization status of the child, possibly reflecting limited access to nutritional counselling and services [43]. Overweight/obesity are also influenced by genetic factors not investigated in this study [44] thus warranting more research. Children whose caregivers had enough time to prepare their food were less likely to be overweight than those who did not have time. Caregivers with sufficient time are more likely to prepare balanced and healthy meals for their children than those without sufficient time. The latter may prefer calorie-dense, nutritionally inferior processed foods, which may increase the risk of being overweight/obese. Children whose birthweight was ≥2.5 kg had high chances of being overweight/obese compared to those with birthweight below 2.5 kg, consistent with another study [45]. However, another study reported higher odds of overweight/obesity among low birthweights [46]. Therefore, there is need for further research to understand the relationship between birthweight and later overweight/obesity risk. Children from households with severe food insecurity had higher odds of being overweight/obese as compared to those from food secure households in agreement with another study [47]. Food insecurity limits food quantity and diversity. Limited food choices can make individuals consume inexpensive high energy or processed foods over healthier ones [48] or take excess food when plenty [49]. Uncertain food access is also thought to trigger changes in metabolic processes leading to excess fat storage as a reserve [50].

**Limitations:** the main limitation of this study is that it employed a cross-sectional design hence the causal relationships between the investigated factors and the malnutrition outcomes could not be established. There is also a bias related to recall, especially for the self-reported explanatory variables. Also, we did not collect data on maternal nutrition and child nutrition-related illnesses such as malabsorption, which limit the understanding of multiple determinants of malnutrition in the study area.

## Conclusion

This study found that childhood malnutrition is prevalent among children aged 6-59 months in Embu County with coexisting overnutrition and undernutrition. A substantial percentage of the children had multiple malnutrition indicators including stunting, wasting, underweight and overweight/obese. Our findings highlight the need for establishment of integrated early childhood development initiatives addressing nutrition, health, and social protection requirements. The integrated programs should encourage age-specific complementary feeding, equal food distribution among 6-23-month-olds, and support for food-insecure families with social protection and livelihood programs. Infection-related malnutrition should also be minimized by regular nutrition screenings, immunization follow-ups and WASH risk mitigation measures. Further studies are needed to understand the socio-economic impacts of malnutrition in the area.

### What is known about this topic

- Childhood malnutrition remains a major public health challenge in developing countries;
- Malnutrition negatively impacts children´s health by disrupting their intellectual and physical growth and increasing risk of infections;
- Childhood malnutrition has several causes encompassing individual, household, societal, environmental and climatic factors.

### What this study adds

- This study presents a high burden of malnutrition with nearly half of children aged 6-59 months experiencing different forms of malnutrition;
- It reveals coexistence of undernutrition and overnutrition within the same context hence the need for multicomponent interventions to address factors associated with malnutrition;
- The findings provide new insights into nutritional status and will inform the design of targeted strategies to mitigate childhood malnutrition.
